# Generative models: an upcoming innovation in musculoskeletal radiology? A preliminary test in spine imaging

**DOI:** 10.1186/s41747-018-0060-7

**Published:** 2018-10-31

**Authors:** Fabio Galbusera, Tito Bassani, Gloria Casaroli, Salvatore Gitto, Edoardo Zanchetta, Francesco Costa, Luca Maria Sconfienza

**Affiliations:** 1 Laboratory of Biological Structures Mechanics, IRCCS Istituto Ortopedico Galeazzi, via Galeazzi 4, 20161 Milan, Italy; 2 Unit of Diagnostic and Interventional Radiology, IRCCS Istituto Ortopedico Galeazzi, via Galeazzi 4, 20161 Milan, Italy; 3 Department of Neurosurgery, Humanitas Clinical and Research Hospital, Via Manzoni 56, 20089 Rozzano, Italy; 4 Department of Biomedical Sciences for Health, Università degli Studi di Milano, via Carlo Pascal 36, 20133 Milan, Italy

**Keywords:** Lumbar vertebrae, Machine learning (deep learning), Magnetic resonance imaging, Neural network (computer), X-rays

## Abstract

**Background:**

Deep learning is a ground-breaking technology that is revolutionising many research and industrial fields. Generative models are recently gaining interest. Here, we investigate their potential, namely conditional generative adversarial networks, in the field of magnetic resonance imaging (MRI) of the spine, by performing clinically relevant benchmark cases.

**Methods:**

First, the enhancement of the resolution of T2-weighted (T2W) images (super-resolution) was tested. Then, automated image-to-image translation was tested in the following tasks: (1) from T1-weighted to T2W images of the lumbar spine and (2) vice versa; (3) from T2W to short time inversion-recovery (STIR) images; (4) from T2W to turbo inversion recovery magnitude (TIRM) images; (5) from sagittal standing x-ray projections to T2W images. Clinical and quantitative assessments of the outputs by means of image quality metrics were performed. The training of the models was performed on MRI and x-ray images from 989 patients.

**Results:**

The performance of the models was generally positive and promising, but with several limitations. The number of disc protrusions or herniations showed good concordance (κ = 0.691) between native and super-resolution images. Moderate-to-excellent concordance was found when translating T2W to STIR and TIRM images (κ ≥ 0.842 regarding disc degeneration), while the agreement was poor when translating x-ray to T2W images.

**Conclusions:**

Conditional generative adversarial networks are able to generate perceptually convincing synthetic images of the spine in super-resolution and image-to-image translation tasks. Taking into account the limitations of the study, deep learning-based generative methods showed the potential to be an upcoming innovation in musculoskeletal radiology.

## Key points

- Deep learning-based generative models are able to generate convincing synthetic images of the spine
- Generative models provide a promising improvement of the level of detail in MRI images of the spine, with limitations requiring further research
- The availability of large radiological datasets is a key factor in improving the performance of deep learning models

## Background

Artificial intelligence and deep learning are ground-breaking technologies that are revolutionising several research and industrial fields. The most notable current uses of deep learning include tasks such as computer vision, pattern recognition of images and classification of complex data, which are increasingly commonly used in sophisticated applications such as robotics, self-driving cars and automated computer programming [1, 2]. Among deep learning technologies, generative models, i.e. computer programs able to generate novel data rather than classify or process existing data, are recently gaining interest due to major technical innovations which are dramatically improving their performance [3–5]. The use of conditional generative adversarial networks (GANs) has recently become a standard for supervised image-to-image translation, i.e. the automatic conversion between two types of images [4]. For example, conditional GANs have been used to colourise black and white pictures and to generate photorealistic images from schematic ones and vice versa [6]. A decisive factor for the current popularity of such technologies is the free availability of most of these computer codes, which are usually written in the popular Python language, as well as of the underlying computational libraries, such as Tensorflow (https://www.tensorflow.org/), Caffe (http://caffe.berkeleyvision.org/), Torch (http://torch.ch/) and PyTorch (https://pytorch.org/). Besides, the training of small- and medium-scale deep learning models does not require specialised hardware, as it can be performed on a standard personal computer equipped with a modern graphics processing unit.

Nowadays, the application of deep learning techniques and generative models in musculoskeletal radiology is still in its infancy. Regarding spine imaging, several studies described the use of simpler machine learning methods such as multilayer perceptron and support vector machines for regression and classification problems, such as the identification of landmarks and the grading of intervertebral disc degeneration [7–9]. More clinically oriented studies—for example, those aimed at the prediction of postoperative complications and patient satisfaction or at the spinal alignment after spine deformity correction surgery—have also been published [10–12]. However, the most recent deep learning techniques, including generative models, did not significantly impact the methods and procedures employed in musculoskeletal radiology so far.

We recently explored the use of conditional GANs for the generation of synthetic radiological images of the spine, namely coronal and sagittal x-rays to be used in in silico trials, i.e. simulated trials aimed to predict the clinical performance of novel implants and surgical techniques before the final validation in a real clinical trial [13]. Nevertheless, generative models likely have the potential to be also employed in the standard diagnostic imaging, by improving the quality of the outputs of the imaging systems as well as by providing additional information which is not accessible in the original images. The aim of the present study was, therefore, to test the potential of generative models in diagnostic musculoskeletal imaging by performing a small set of clinically relevant benchmark cases in the field of spine imaging.

## Methods

Ethical committee approval for this retrospective study has been obtained and patients’ informed consent was waived. All magnetic resonance imaging (MRI) examinations were performed using one of two 1.5-T systems installed at our institution (Avanto, gradient strength 45 mT/m, slew rate 200 T/m/ms; or Espree, gradient strength 33 mT/m, slew rate 170 T/m/ms; Siemens Medical Solutions, Erlangen, Germany).

Two distinct potential applications of conditional GANs in the field of spinal imaging were tested in the present work. First, the enhancement of the resolution of T2-weighted (T2W) images (super-resolution) was performed. Then, the automated translation between different imaging modalities, referred to in the following paragraphs as virtual multimodal imaging, was attempted. The sizes of the training, validation and test datasets for each task are summarised in Table 1.

**Table 1:** Sizes of training, validation, and test datasets for all the considered tasks

Task	Training	Validation	Test
Super-resolution	767	192	30
T1-weighted to T2-weighted	767	192	30
T2-weighted to T1-weighted	767	192	30
T2-weighted to STIR	284	71	30
T2-weighted to TIRM	305	77	30
Sagittal x-ray to T2-weighted	363	91	30

### Conditional GANs and Pix2pix

Standard, i.e. unconditional, GANs consist of two components: a model, named the discriminator, which is trained to discriminate whether an image is real or fake; and a generator, which learns to create realistic new data trying to fool the discriminator, with the final aim of ‘making the output indistinguishable from reality’ [4, 5]. In other words, the discriminator teaches the generator how to create a realistic output, while simultaneously learning how to discriminate real and fake data. Unconditional GANs have been used for image-to-image translation problems by designing a loss function, such as L1 or L2 regression or more complicated application-specific functions, to link the output of the GANs to the input [14, 15]. Regression-based loss functions consider each pixel in the output as independent, not accounting for its relation to the surrounding parts of the image, and thus favours the optimisation on a pixel-to-pixel basis rather than of the image structure as a whole.

Whereas in unconditional GANs the generator does not see the input image, in conditional GANs both generator and discriminator receive as input the image to be translated [4, 16]. The loss function of conditional GANs is learned, rather than predefined. Research showed that these architectural improvements provided clearly superior performances with respect to GANs using only traditional regression loss functions [4], which tend to produce blurry outputs [17]. Indeed, conditional GANs now constitute the state-of-the-art for image-to-image translation.

In this work, we used pix2pix [4], the original formulation of conditional GANs for image-to-image translation, in its Tensorflow implementation (available at https://github.com/affinelayer/pix2pix-tensorflow), which closely follows the original implementation published by the authors based on Torch (https://github.com/phillipi/pix2pix). This port does not include the ‘U-Net’ architecture with skips for the generator [18] described in the original paper [4], and allows for combining the conditional GAN objective function with a L1 regression loss, as desired by the user. As suggested by Isola et al., we used a combination of loss weights (1 for the conditional GAN loss, 100 for the L1 loss) which were shown to ensure a sharp output while limiting the occurrence of visual artifacts. Since the original implementation only supports images with a size of 256 × 256 pixels, for the task involving images of 512 × 512 pixels we enlarged the encoder–decoder networks of both the generator and the discriminator to allow the processing of such images. All models were trained from scratch on a Linux workstation equipped with a NVIDIA Titan Xp GPU. Source codes and pre-trained models used and developed in this study are publicly available at https://goo.gl/xAgkbd.

### Super-resolution

From a large set of high quality T2W images of the lumbar spine available in the picture archive and communication system (PACS) of IRCCS Istituto Ortopedico Galeazzi, 989 midsagittal images with a size of 512 × 512 pixels were collected. All images were linearly down-sampled to a size of 128 × 128 pixels and then linearly re-upscaled to 512 × 512 pixels to obtain a low-resolution image containing no fine details. Conditional GANs were then trained to reconstruct a sharp image of 512 × 512 pixels, starting from the corresponding low-resolution one.

### Virtual multimodal imaging

The same technique used for super-resolution, i.e. the standard image-to-image translation by means of conditional GANs, was tested in the automated translation between different imaging modalities. Five tasks were attempted with this technique: (1) the translation from T1-weighted (T1W) to T2W images of the lumbar spine; (2) the reverse translation, i.e. from T2W to T1W images; (3) from T2W to short tau inversion recovery (STIR) images; (4) from T2W to turbo inversion recovery magnitude (TIRM) images; (5) from sagittal standing x-ray projections to T2W images. For the first four tasks, the training datasets were created based on a database of 989 patients subjected to T1W and T2W imaging of the lumbar spine for the investigation of low-back pain available in our PACS. In the image database, STIR imaging was available for 385 individuals, while TIRM images were acquired for 412 patients. All images had a size of 256 × 256 pixels. To maximise the size of the training datasets, in addition to the midsagittal images of the lumbar spine, the adjacent images were also included, for a total of three images for each patient.

**Fig. 1:**
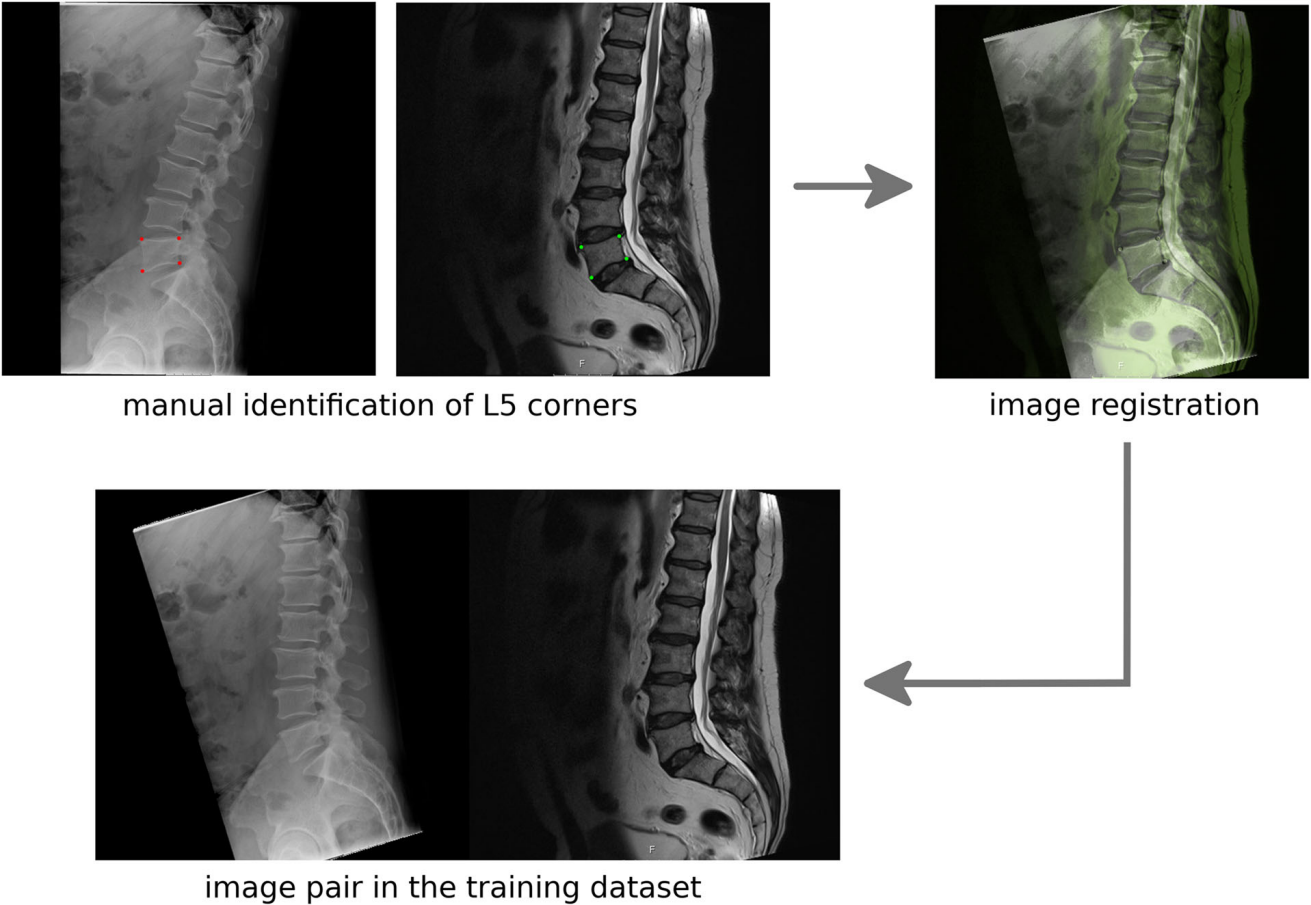
The manual registration procedure used for the alignment of the sagittal x-ray projections and the midsagittal T2W MRI scans. First, the vertebral corners of L5 are manually identified on both images. Then, a rigid registration is performed and the pair of registered images is added to the training dataset

For the fifth translation task aimed at the generation of T2W images from sagittal standing x-ray projections, 484 patients for whom images acquired in both modalities were available in our PACS were identified. After resizing all images to 256 × 256 pixels, all planar radiographic projections were registered to the corresponding midsagittal MR image, so that the corners of the vertebral body of L5 were approximately in the same location in both images (Fig. 1). To this aim, an in-house C++ computer program which allows for the manual identification of the corner points of L5 and then performs a Euclidean transformation of the images was developed and used to align each couple of images for the 484 patients. Subsequently, a generative model able to translate the radiographs to the registered T2W midsagittal MR images was trained.

### Quantitative validation

All image-to-image translation models were tested on 30 images for which the ground truth, called ‘target’, was available. The quality of the generated outputs was assessed by means of commonly employed metrics such as the mean squared error (MSE), the peak signal-to-noise ratio (PSNR), the structural similarity index (SSIM) [19] and the visual information fidelity in pixel domain (VIPF) [20], taking as reference the target image. Furthermore, the cumulative probability of blur detection (CPBD) [21], which does not require a reference image for its calculation, was determined to provide a quantitative assessment of the image sharpness. MSE, PSNR and SSIM were evaluated with the implementations available in scitik-image (https://scikit-image.org/), whereas scikit-video (http://scikit-video.org) was used for the calculation of VIPF. CPBD was determined by using the Python module available at https://ivulab.asu.edu/software/cpbd. For the super-resolution task, the quality of the generated images was determined by means of PSNR, SSIM, VIPF and CPBD. As a baseline for comparison with PSNR, SSIM and VIPF, the down-sampled images of 128 × 128 pixels were upscaled to 512 × 512 pixels by both linear and cubic resampling. The other image-to-image translations tasks were quantitatively assessed by means of MSE, PSNR and SSIM.

### Clinical evaluation

A musculoskeletal radiologist with four years of experience evaluated the synthetic images of 30 anonymised and randomly selected patients. Specifically, the number of disc protrusions or herniations and the presence of artifacts were assessed in the images generated with super-resolution. The presence of L4-L5 degenerative disc disease, L5-S1 degenerative disc disease and L4-S1 Modic-type endplate changes [22] was assessed for the T1W-to-T2W translation and the reverse translation, as well as T2W-to-STIR and T2W-to-TIRM translation tasks. Finally, the presence of abnormal numbering and body compression fractures of the lumbar vertebrae were assessed on the x-ray to T2W translated images. After a delay of 14 days aimed at minimising the recall of given studies, the same musculoskeletal radiologist evaluated the target images (i.e. the native images) of the same patients. Specifically, the features previously evaluated in the synthetic images, except the super-resolution task-related artifacts, were assessed. Concordance between the synthetic and target images was calculated using κ-statistics and interpreted as follows: < 0.20, poor; 0.21–0.40, fair; 0.41–0.60, moderate; 0.61–0.80, good; 0.81–1.00, excellent [23].

## Results

**Fig. 2:**
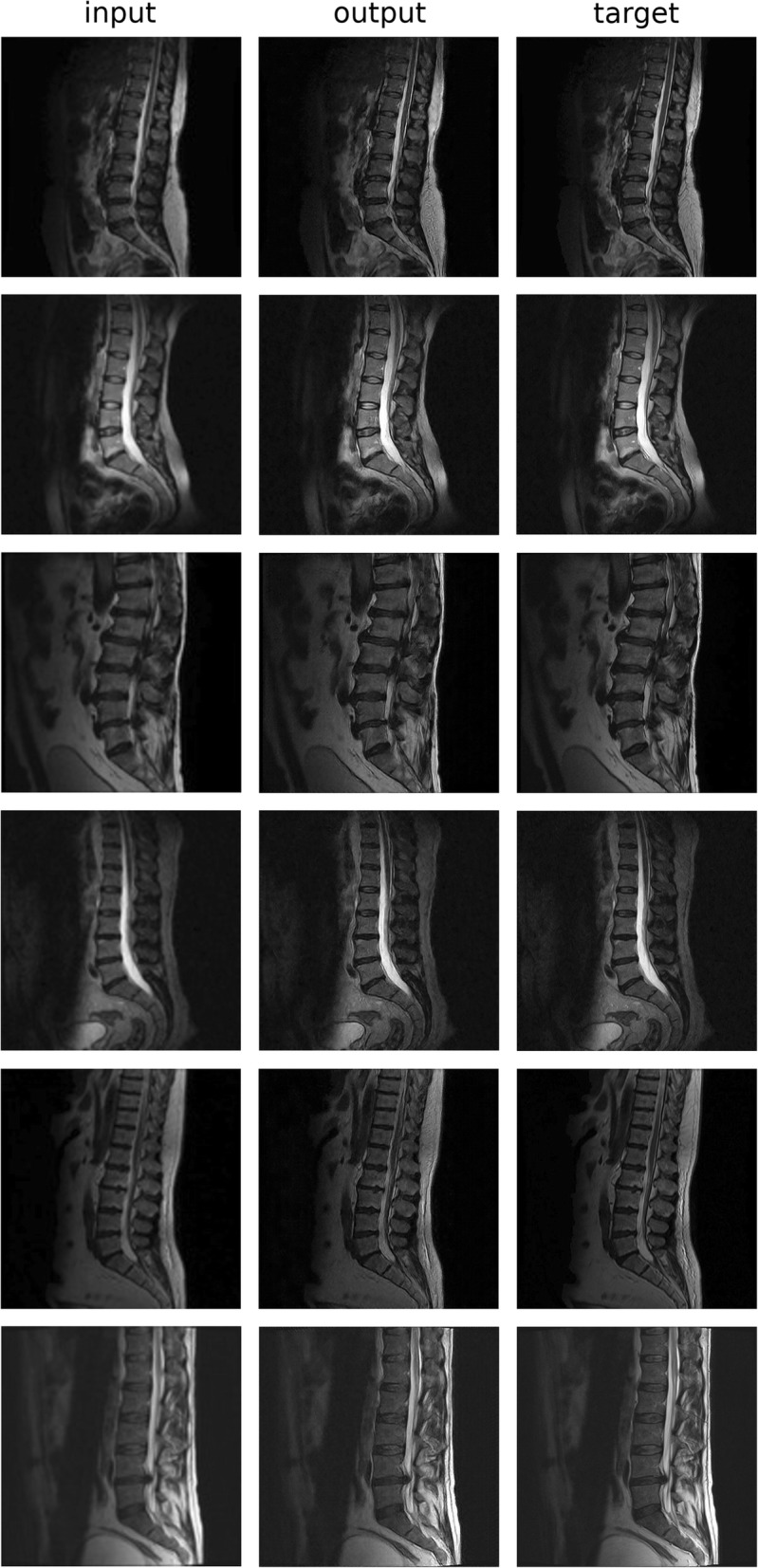
Six examples of the use of GANs for the super-resolution task. input: original low-resolution image; output: output of the generative model; target: original high-resolution image. Multiple parallel edges similar to truncation artifacts are visible in proximity of high-contrast boundaries

**Fig. 3:**
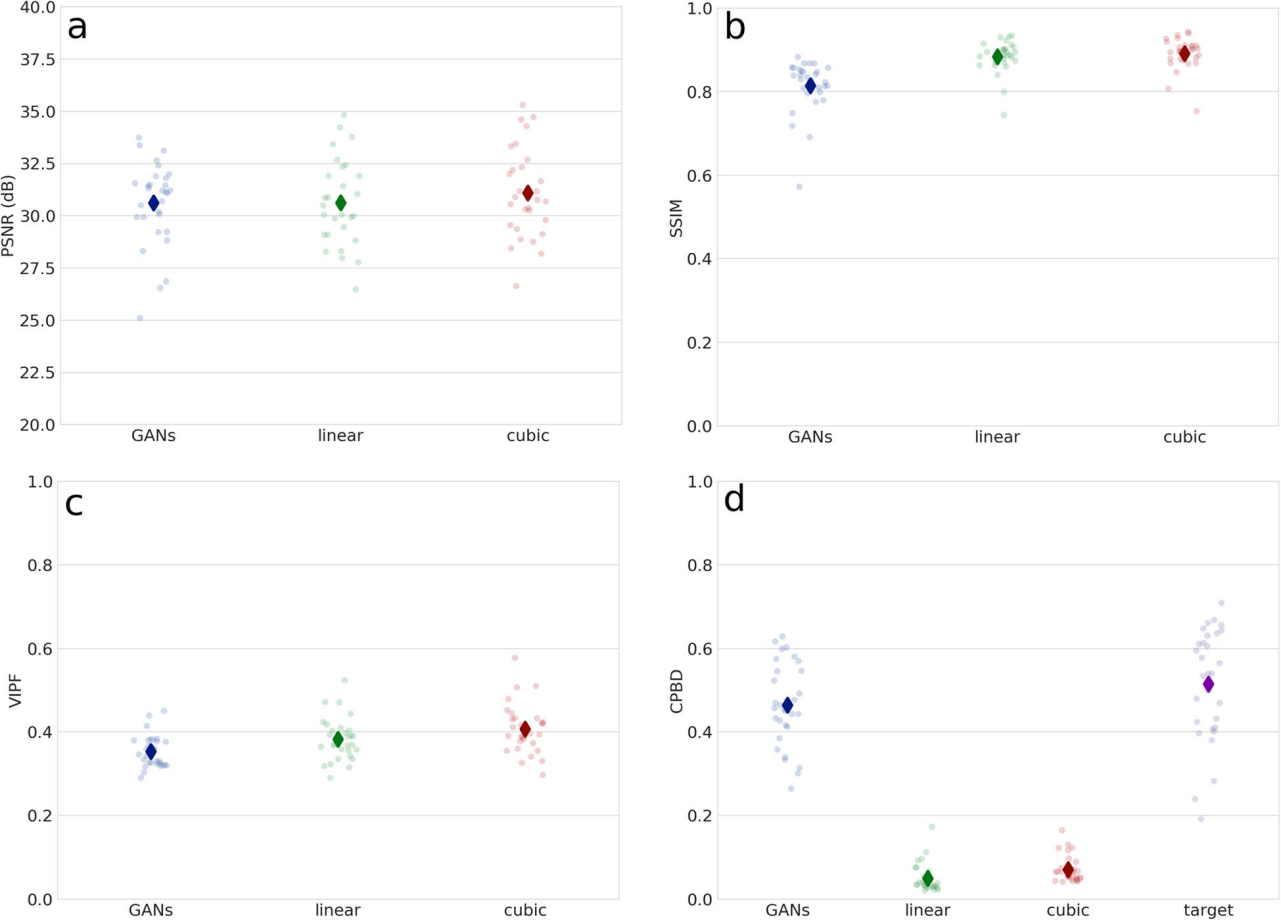
Quantitative comparison among the outputs of GANs (GANs), linear resampling (linear) and cubic resampling (cubic) by means of the metrics PSNR (**a**), SSIM (**b**), VIPF (**c**) and CPBD (**d**). For CPBD, the value of the metric calculated for the target image (target) is also shown for the sake of comparison

Conditional GANs improved image sharpness and visible details in the T2W images in the super-resolution task (Fig. 2). The generative model was able to add realistic details which were not visible in the low-resolution images. Additional visual information with a potential to be clinically relevant can be easily depicted in the intervertebral discs (including protrusions), in the spinal cord and even in the adipose tissue. However, the super-resolution images are affected, in a variable measure, by inaccuracies visually resembling truncation artifacts. Unexpectedly, the quantitative evaluation of the quality of the outputs, as compared with standard linear and cubic upsampling, did not result in a better performance of the GANs based on PSNR, SSIM and VIPF (Fig. 3). Nevertheless, the generative models were superior in restoring the image sharpness with respect to the ground truth, based on the assessment with the CPBD metric.

**Fig. 4:**
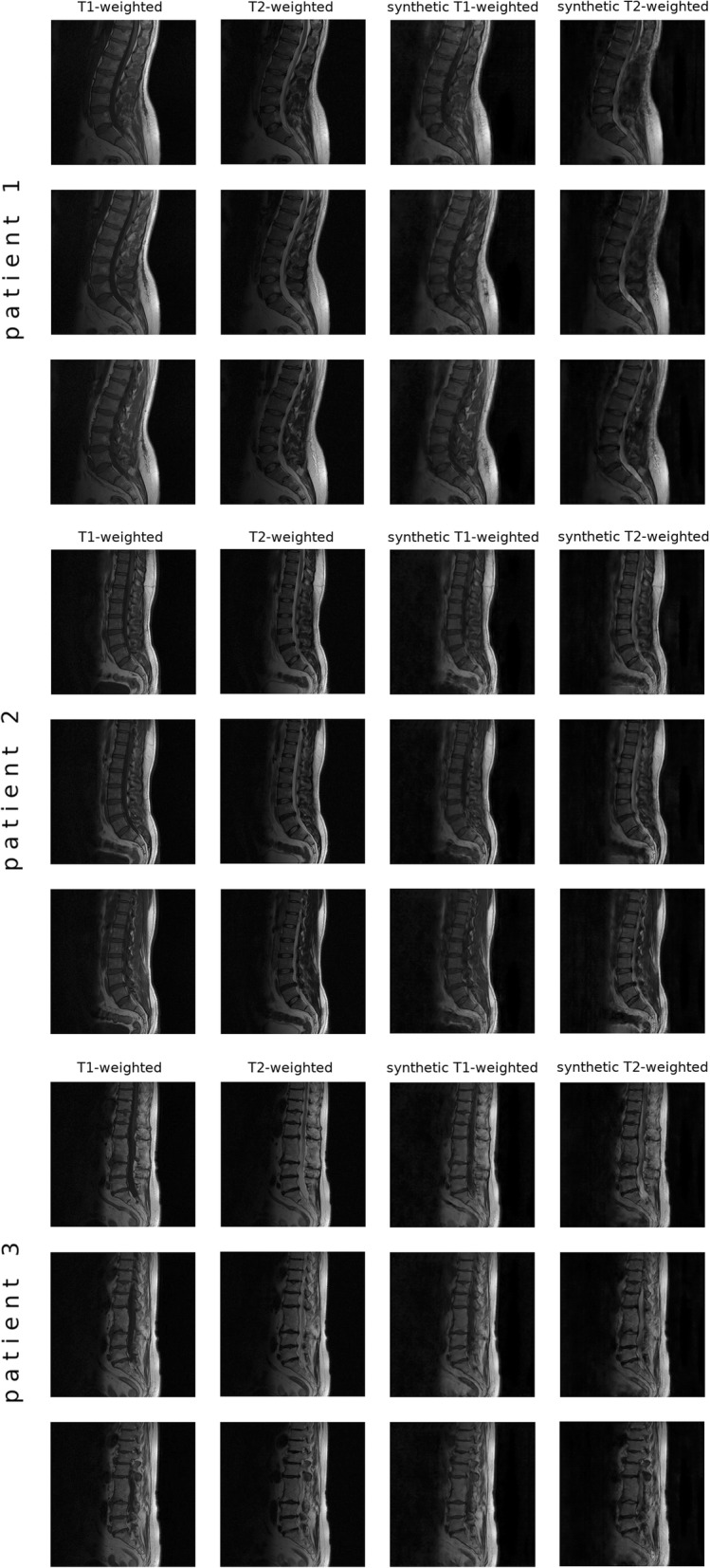
Representative results (three slices for three different patients) of the translation from T1W to T2W MRI and vice versa. In the first patient, Type II Modic changes were correctly represented in both synthetic images, whereas for the Type I change in the third patient the synthetic T2W image showed a low signal instead of a high one. The L4-L5 disc protrusion of the second patient was accurately represented in the synthetic T2W image

In the tasks related to virtual multimodal imaging, the performance of the conditional GANs can be judged as generally positive and promising; however, a deeper analysis of the outputs revealed several limitations. The translation between T1W and T2W images and vice versa (Fig. 4) demonstrated that the used generative model was able to deal well with the general features and differences between the two imaging modalities, such as the distinct grey levels in the spinal cord and in the nucleus pulposus of the intervertebral discs. However, healthy discs with high water content tended to have a slightly brighter representation in the nucleus, even in the synthetic T1W MR images, in contrast with the original images in which discs have a rather homogeneous grey level independently on the disc height or degeneration degree. On the other side, synthetic T2W images clearly depicted protruded discs and the correspondent compression of the spinal cord, which were less evident in the T1W images used as inputs. Degenerated discs tended to be correctly darker than healthy ones in synthetic T2W images. As expected, the generative model was generally not able to deal correctly with Modic changes; in general, the signal alteration found in the original image was directly translated to the synthetic image, i.e. whereas type II changes were correctly represented, type I changes appeared similar to type III ones in the T1W-to-T2W translation.

**Fig. 5:**
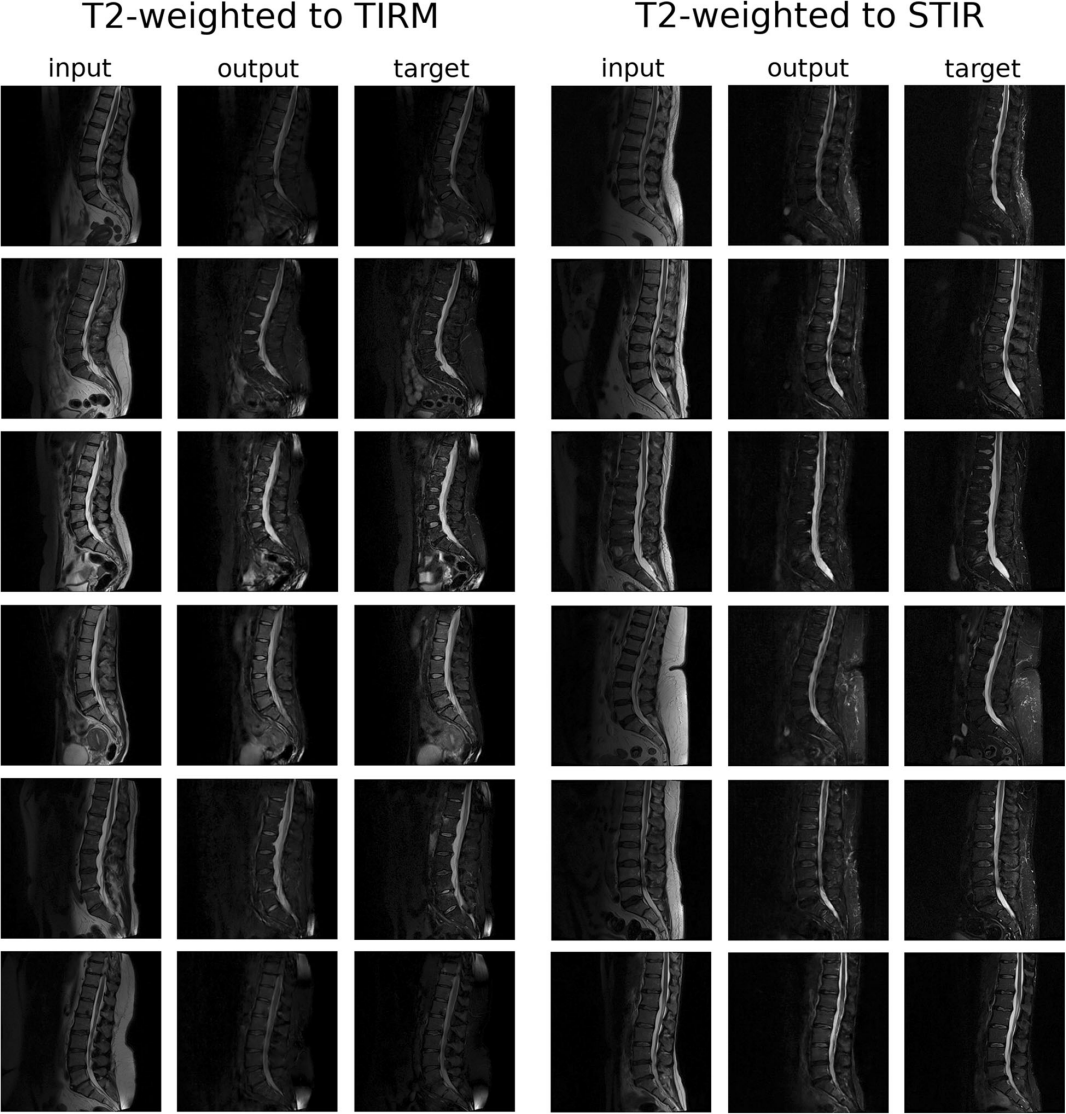
Representative results of the translation from T2W MRIs to TIRM (*left*) and to STIR (*right*) images. input: original T2W image; output: TIRM/STIR image created by the generative model; target: original TIRM/STIR image. Images from six exemplary patients are shown for each translation

From a qualitative point of view, the performance of the conditional GANs in translating T2W scans to TIRM and STIR scans appeared to be excellent (Fig. 5). Similar to the T1W-to-T2W translation, the model was capable of dealing well with the general features of the images, such as the fat suppression and signal alterations in the pathologic vertebrae.

**Fig. 6:**
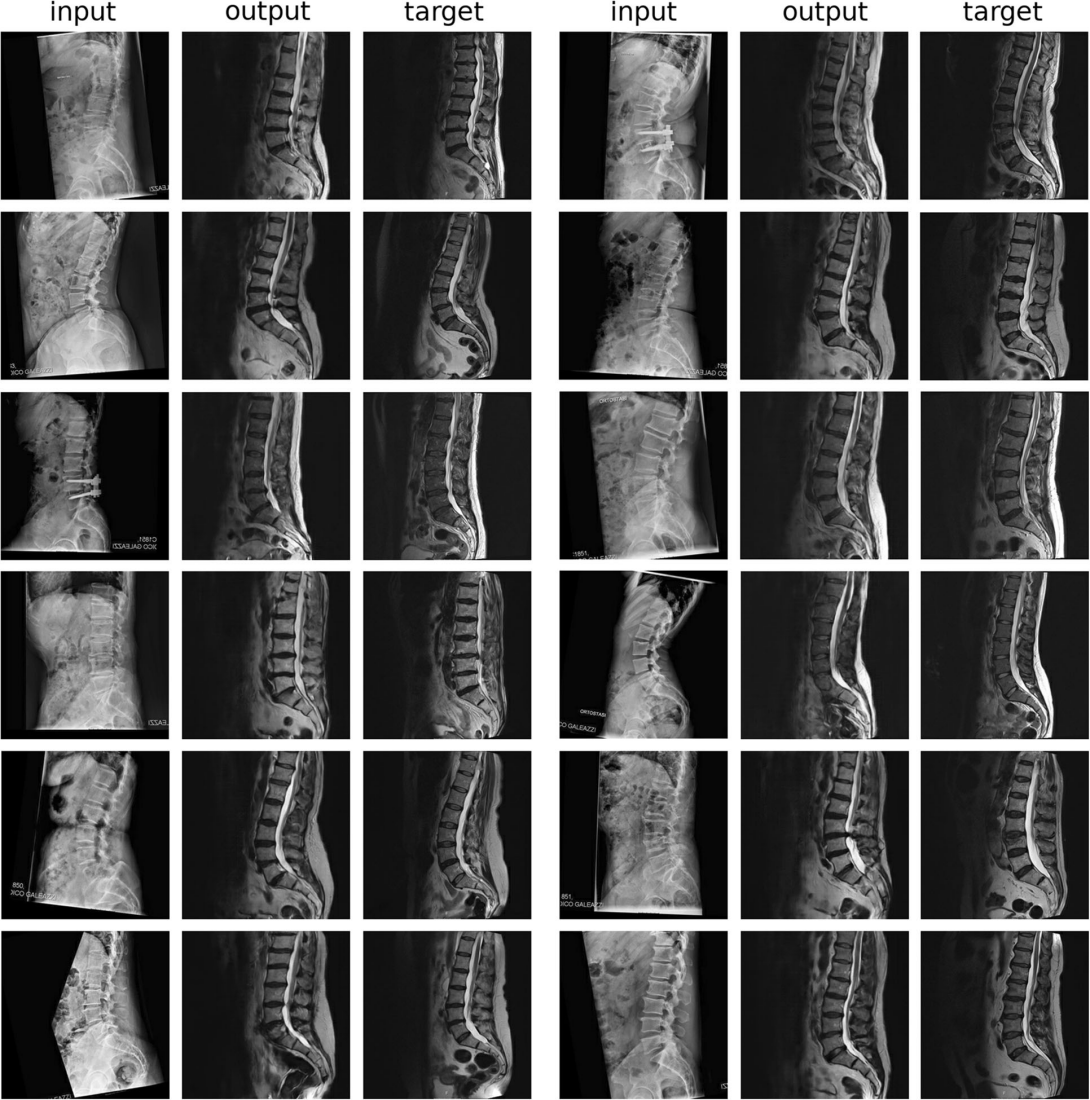
Representative results (12 patients) of the translation from sagittal x-ray projections to T2W midsagittal MRI scans. input: original x-ray image, registered to match the alignment of the original MRI; output: synthetic T2W midsagittal MRI; target: original T2W midsagittal MRI. A few gross errors in the anatomy can be identified: the L1 in the first and fourth patients on the *left*; the sacrum in the first patient on the *right*; the thoracolumbar junction in the third patient on the *right*; L1 and the sacrum in the fourth patient on the *right*

The challenging translation from sagittal x-ray projections to T2W midsagittal MRI scans provided very interesting results which included a few evident errors in the depiction of the anatomy of the patient, but on the other hand highlighted the enormous potential of generative models (Fig. 6). Indeed, the synthetic MRI images showed in numerous cases incorrect anatomies, such as unrealistically long or short vertebral bodies, especially in regions not clearly visible on the x-ray projections, such as the thoracolumbar junction. Nevertheless, the conditional GANs were able to create realistically looking MR images which include a basically correct depiction of the spinal cord, of the intervertebral discs and of the layer of adipose tissue on the back. The lower lumbar lordosis due to the different posture (standing in the sagittal x-ray examination versus supine in MRI) was also realistically captured.

**Fig. 7:**
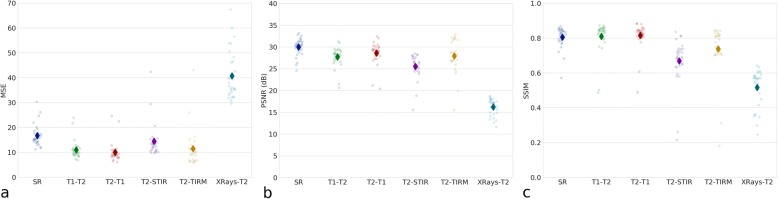
Quantitative evaluation of the quality, based on different metrics (MSE (**a**), PSNR (**b**) and SSIM (**c**)) of the various image-to-image translation tasks: super-resolution (SR), T1W to T2W MRI images (T1-T2), T2W to T1W images (T2-T1), T2W to STIR images (T2-STIR), T2W to TIRM images (T2-TIRM), sagittal standing x-ray projections to T2W images (Xrays-T2)

The quantitative evaluation of the image quality with respect to the ground truth revealed a consistent pattern among the three considered metrics MSE, PSNR and SSIM (Fig. 7). The translation of T1W images into T2W images and vice versa gave the most accurate results, followed by the translation from T2W to TIRM and STIR images respectively. As expected, the translation of sagittal x-ray projection to MRI consistently resulted in the worst quantitative performance.

With regard to clinical evaluation, the number of disc protrusions or herniations showed good concordance (κ = 0.691) between the native images and those generated with super-resolution. The median percentage of truncation artifacts amounted to 20%. Table 2 reports the concordance between the native images and those generated with virtual multimodal imaging. Moderate-to-excellent concordance was found when translating T2W to STIR and TIRM images, while the agreement was poor when translating x-ray projections into T2W images.

**Table 2:** Concordance (expressed as κ-value) between the native images and those generated with virtual multimodal imaging

	L4-L5 disc disease	L5-S1 disc disease	L4-S1 Modic-type endplate changes
T1-weighted to T2-weighted	0.455 (moderate)	0.221 (fair)	0.406 (moderate)
T2-weighted to T1-weighted	0.086 (poor)	0.270 (fair)	0.286 (fair)
T2-weighted to STIR	0.842 (excellent)	1.000 (excellent)	0.592 (moderate)
T2-weighted to TIRM	0.842 (excellent)	0.933 (excellent)	0.691 (good)
	Lumbar vertebrae number	Lumbar vertebral body fractures	
Sagittal x-ray to T2-weighted	0.065 (poor)	0.051 (poor)	

## Discussion

In this paper, we explored the use of generative models, namely conditional GANs, for the creation of synthetic images of the spine and for the improvement of the quality of existing images. Despite several inaccuracies in the outputs, including some evident mistakes such as in the number of vertebrae, the general performance of the method should be judged as positive and very promising in light of future applications. As a matter of fact, no similar results have ever been reported in the available literature, neither with deep learning-based methods nor with other techniques. A paper describing a similar approach was aimed at the generation of synthetic images to be used in in-silico trials [13], rather than to proper clinical applications. Recent research highlighted the potential of conditional GANs for other radiological tasks, such as the improvement of the quality of low-dose positron emission tomography imaging [24] and noise reduction in low-dose computed tomography (CT) [25].

Taking into account the constant, fast advance of deep learning techniques for image synthesis and the large number of options for the technical refinement of the methods discussed in the present work, we foresee an enormous improvement of the quality of the generated data in the next future. It should be noted that the present work was not aimed at developing novel techniques for radiological image synthesis, but rather at exploring the potential of the methods currently available, knowing that research targeted specifically to radiology may provide even better results soon.

Although a preliminary quantitative assessment of the validity of the outputs of the generative models has been provided, the current work should still be intended as an exploratory proof of concept. As a matter of fact, the actual value of any innovative technique should be evaluated based on the impact that it can give on the practical applications rather than simply on the technical evaluation of its outputs such as the one here reported. Nevertheless, such a ground-breaking method opens new perspectives, in terms of potential applications, which still need to be explored.

Concerning spine imaging, possible clinically relevant uses include the grading of disc degeneration from planar x-ray imaging whenever an MRI scan is not available, the correction of the spinal shape due to different postures (e.g. standing versus supine), the improvement of the resolution of images acquired with low-field MRI scanners and the prediction of the effect of loading on the soft tissues, for example for the study of disc protrusions under loading. Additionally, virtual multimodal imaging may be ultimately integrated in PACS clients and Digital Imaging and COmmunications in Medicine (DICOM) viewers, to allow for a preliminary analysis of patients for which incomplete data are available. In case of diagnostic CT or MRI exams in which only a few slices have been acquired, the use of generative models may allow for synthetic re-slicing and thus high-quality visualisation also in the non-acquired orientations. Besides, we may foresee an MRI protocol for spine imaging using a single sequence or three sequences with different weighting composed by few slices. From that, such a model may generate a full set of MRI sequences, thus remarkably reducing exam duration and MR system occupation. A similar approach has been recently reported on the knee, although based on a different technology [26].

For diagnostic purposes, the synthesis of a new image may not always be the optimal solution to achieve an improvement of the sensitivity and/or specificity of the diagnosis. Indeed, if the information required for the clinical evaluation is already available in the original image (e.g. information about intervertebral disc degeneration in a planar x-ray projection), the generation of a complete synthetic MRI scan showing the degenerative features of the disc may be deemed as superfluous for the diagnosis and grading of the disorder. Although we believe that several practical cases in which image synthesis can provide a clear benefit to musculoskeletal imaging, even from the clinical point of view, will definitely emerge as time goes by, it should be noted that simpler solutions may still be clinically advantageous for specific applications.

To our knowledge, the only application in which synthetic imaging data are nowadays used is MRI-only radiation therapy treatment planning [27]. In conventional radiation treatment, both MRI and CT images are acquired and used for planning and verification of the patient positioning. The simultaneous use of both imaging modalities requires a registration step, which introduces a systematic error not negligible from a clinical point of view. To eliminate it, an MRI-only workflow has been introduced, in which a synthetic CT is generated based on the MRI data. Various algorithms have been proposed for the generation of synthetic CTs, ranging from simple override techniques [28] to atlas-based ones [29, 30] and finally to sophisticated statistical models [31, 32]. The potential of conditional GANs for this specific application, possibly in combination with other consolidated approaches, is evident.

Although affected by artifacts, the super-resolution task provided very good results from a perceptual point of view. As a matter of fact, super-resolution is not a new concept and several algorithms have been proposed [33], with a special focus on MRI [34, 35]. Since the detection of small lesions may challenge even modern MRI scanners, this topic gains a specific clinical relevance. With respect to the classical MRI super-resolution techniques which rely on specific acquisition and reconstruction techniques, deep learning-based super-resolution can be applied as post-processing any time after the image reconstruction, with obvious advantages. Besides, generative models may add details not directly visible in the original images, based only on patterns found in similar patients, such as a specific shape, grey level or texture. The possible impact of these added details on the future clinical applications, either positive or negative, should not be neglected, since they can lead to misdiagnosis if they refer to non-existent pathological features. The clinical evaluation conducted in this study highlighted that such artifacts indeed affected the outputs of the generative models, such as the number of vertebrae visible in the generated images and the occurrence of fractures in the translation from x-ray projection to T2W MRI. It should be noted that visual artifacts may be avoided or reduced by optimising the loss function of the model, for example by increasing the weight of the L1 regression with respect to the conditional GAN loss or by introducing a L2 loss term. Besides, such optimisation may benefit the quality metrics findings, whose results were not up to our expectations. As a matter of fact, the weights in the loss function used in this study arguably favoured sharpness over similarity to the target, with a clear negative impact on the metrics. These aspects were not investigated in the present paper, in which the weights of the two terms of the objective functions were kept fixed but need to be further analysed in future studies.

The results of image-to-image translation tasks also highlighted the potential of the generative framework. Similar to super-resolution, the novel methods can be applied in post-processing, since they do not require any modification to the acquisition and reconstruction stages. In this respect, generative models substantially differ from another documented MRI technique, synthetic MRI (SyMRI), providing a similar output, i.e. generating synthetic contrast-weighted images after the acquisition of the data [36–38]. Indeed, SyMRI dictates the use of a specific protocol creating a raw image which can then be post-processed to generate T1W, T2W and proton density maps and cannot be used on existing datasets acquired with other MRI protocols. It should be noted that, despite the generally convincing visual appearance of the translated images, a more extensive validation as well as an optimisation of the technique for the specific radiological applications are necessary before any clinical use of the novel techniques. The validation tests should address directly the specific clinical questions for which sequences such as STIR and TIRM are used, such as the diagnosis of soft-tissue tumours [39] and osteomyelitis [40], rather than being limited to a general evaluation of the quality of the synthetic images.

Due to its preliminary nature and its novelty, the present work suffers from several limitations, the most important of which is indeed the limited extent of the clinical validation. Furthermore, we decided to use an available implementation aimed to general image-to-image translation, without customising it to the specific application. As mentioned above, even simple optimisations such as the adjustment of the weights in the loss function may have a positive impact on the quality of the results. Another limitation pertains to the limited size of the training datasets, which has been constrained by practical issues related to the availability and traceability of the images. We expect that increasing the number of images constituting the training data would involve a major improvement in the quality of the outputs.

## Conclusions

In conclusion, this proof of concept study showed that conditional GANs are able to generate perceptually convincing synthetic images of the spine, in super-resolution and image-to-image translation tasks. With respect to other methods providing analogous outputs, conditional GANs do not require specific acquisition and reconstruction techniques, and they can be employed in post-processing to any existing images. Although a clinical validation is still missing, we believe that conditional GANs, and deep learning-based generative methods in general, have the potential to be an upcoming innovation in musculoskeletal radiology.

## Abbreviations

CPBD: Cumulative probability of blur detection
CT: Computed tomography
GANs: Generative adversarial networks
MRI: Magnetic resonance imaging
MSE: Mean square error
PSNR: Peak signal-to-noise ratio
SSIM: Structural similarity
STIR: Short tau inversion recovery
T1W: T1-weighted
T2W: T2-weighted
TIRM: Turbo inversion recovery magnitude
VIPF: Visual information fidelity in pixel domain
